# Alcohol-Related Deaths of US Health Care Workers

**DOI:** 10.1001/jamanetworkopen.2024.10248

**Published:** 2024-05-08

**Authors:** Mark Olfson, Candace M. Cosgrove, Melanie M. Wall, Carlos Blanco

**Affiliations:** 1 Department of Psychiatry, College of Physicians and Surgeons, Columbia University and the New York State Psychiatric Institute, New York, New York; 2 Census Bureau, Mortality Research Group, Suitland, Maryland; 3 Division of Epidemiology, Services, and Prevention Research, National Institute on Drug Abuse, Rockville, Maryland

## Abstract

This cohort study investigates the risk of alcohol-related death among US health care workers compared with non–health care workers.

## Introduction

Despite an increase in alcohol-related deaths in the US over the last 2 decades,^1,2^ little is known about alcohol-related mortality risks among physicians^3^ and other health care workers. Compared with non–health care workers, health care workers have increased drug overdose death risks.^4^ An occupational liability to substance use or to risks of painful injuries and access to controlled substances may explain these findings. Only the first hypothesis would contribute to an increased risk of alcohol-related mortality. We estimated risks for alcohol-related deaths among US health care workers compared with non–health care workers.

## Methods

This cross-sectional study was deemed exempt from review and the need for informed consent by the institutional review board of the New York State Psychiatric Institute owing to the use of only deidentified data. We followed the STROBE reporting guideline.

The 2008 American Community Survey (ACS) was a cross-sectional, nationally representative survey of approximately 2.9 million addresses with a 97.9% response rate. Data were linked to National Death Index records from 2008 to 2019.^5^ After excluding unemployed individuals and those younger than 26 years, the cohort included 1 838 000 individuals. Alcohol-related underlying or contributing causes of death were identified (eTable 1 in Supplement 1). Health care workers included (1) registered nurses, (2) support workers, (3) technicians, 4) social and behavioral workers, (5) other diagnosing or treating clinicians (eg, dentists), and (6) physicians (eTable 2 in Supplement 1). Event time was from ACS administration to alcohol-related death, death from other causes, or December 31, 2019, whichever came first, with follow-up between 11 and 12 years for participants still living.

Analyses were performed in SAS, version 9.4 (SAS Institute Inc). Unadjusted and age- and sex-standardized alcohol-related death rates per 100 000 person-years with 95% CIs were calculated (2-sided *P* < .05 indicated statistical significance). Cox proportional hazards regression models estimated alcohol-related death hazard ratios for the 6 health care groups adjusted for age, sex, race and ethnicity, marital status, educational level, and income. Race and ethnicity were included as covariates given known disparities in alcohol-related deaths among US racial and ethnic groups. We applied ACS weights.^6^

## Results

The median age of the overall cohort was 44 (IQR, 35-53) years; 47.7% were female and 52.3% were male. Group sociodemographic characteristics are presented in Table 1. Unadjusted alcohol-related death rates per 100 000 person-years were significantly lower for each health care worker group than for non–health care workers. Following age and sex standardization, only physicians (4.2) and other diagnosing or treating clinicians (11.6) had significantly lower alcohol-related death rates than non–health care workers (18.1). Controlling for the other sociodemographic characteristics, alcohol-related mortality hazards did not significantly differ between each health care worker group and non–health care workers (Table 2).

**Table 1.** Sociodemographic Characteristics of Health Care and Non–Health Care Occupational Groups

Characteristic	Occupational group, % (95% CI)^a^						
	Registered nurses (n = 42 000)	Health care support workers (n = 39 000)	Health technicians (n = 32 500)	Social or behavioral health workers (n = 27 000)	Other diagnosing or treating clinicians (n = 22 500)	Physicians (n = 13 000)	Non–health care workers (n = 1 666 000)
Age, y							
26-35	32.4 (31.8-32.9)	25.8 (25.3-26.4)	26.7 (26.2-27.3)	25.0 (24.5-25.7)	25.8 (25.2-26.5)	26.2 (25.3-27.0)	25.2 (25.1-25.3)
36-45	19.5 (19.0-19.9)	16.3 (15.8-16.7)	15.7 (15.2-16.1)	21.2 (20.7-21.8)	18.5 (17.9-19.0)	24.6 (23.7-25.4)	28.0 (27.9-28.1)
46-55	21.4 (20.9-21.9)	30.0 (29.4-30.5)	29.6 (29.3-30.3)	28.7 (28.0-29.4)	27.4 (26.7-28.2)	21.5 (20.7-22.4)	27.4 (27.4-27.5)
>55	26.7 (26.2-27.3)	27.9 (27.4-28.5)	28.0 (27.4-28.6)	25.1 (20.7-21.8)	28.2 (27.5-29.0)	27.7 (26.8-28.6)	19.4 (19.3-19.4)
Sex							
Female	91.1 (90.7-91.4)	89.6 (89.2-89.9)	78.3 (77.7-78.8)	72.5 (71.9-73.2)	60.9 (60.1-61.6)	32.4 (31.4-33.3)	43.4 (43.3-43.5)
Male	8.9 (8.6-9.3)	10.4 (10.1-10.8)	21.7 (21.2-22.3)	27.5 (26.8-28.1)	39.1 (38.4-39.9)	67.6 (66.7-68.6)	56.6 (56.5-56.7)
Race and ethnicity							
Hispanic	4.3 (4.1-4.6)	13.3 (12.9-13.8)	7.7 (7.4-8.1)	10.4 (9.9-10.8)	5.0 (4.6-5.3)	5.7 (5.2-6.2)	14.0 (13.9-14.1)
Non-Hispanic Black	10.1 (9.7-10.5)	26.7 (26.1-27.3)	15.5 (14.9-16.0)	19.5 (18.9-20.1)	6.3 (5.8-6.7)	5.3 (4.8-5.8)	10.1 (10.0-10.2)
Non-Hispanic White	76.2 (75.7-76.7)	53.4 (52.8-54.0)	69.9 (69.2-70.5)	65.4 (64.7-66.2)	79.2 (78.5-79.9)	70.2 (69.2-71.1)	69.4 (69.4-69.5)
Other^b^	9.4 (9.0-9.7)	6.5 (6.2-6.8)	6.9 (6.5-7.2)	4.7 (4.4-5.0)	9.5 (9.1-10.0)	18.8 (18.0-19.6)	6.4 (6.4-6.5)
Marital status							
Married	66.8 (66.2-67.4)	50.5 (49.9-51.2)	60.1 (59.4-60.7)	56.3 (55.5-57.0)	71.4 (70.6-72.1)	77.7 (76.8-78.6)	63.2 (63.1-63.3)
Separated or divorced	18.0 (17.6-18.5)	23.1 (22.6-23.6)	19.3 (18.8-19.9)	17.9 (17.3-18.5)	11.6 (11.1-12.10	7.8 (7.2-8.4)	15.8 (15.8-15.9)
Widowed	2.6 (2.4-2.8)	4.1 (3.9-4.3)	2.5 (2.3-2.8)	2.3 (2.1-2.5)	1.3 (1.1-1.4)	1.0 (0.8-1.3)	2.2 (2.2-2.2)
Never married	12.5 (12.1-12.9)	22.3 (21.7-22.8)	18.1 (17.5-18.6)	23.6 (22.8-24.2)	15.8 (15.2-16.4)	13.4 (12.7-14.2)	18.8 (18.7-18.8)
Educational level							
Less than high school	0.4 (0.3-0.5)	11.4 (11.0-11.8)	1.6 (1.4-1.7)	1.4 (1.2-1.6)	0.7 (0.5-0.8)	0.2 (0.2-0.3)	10.4 (10.4-10.5)
High school or GED	1.7 (1.5-1.8)	33.6 (33.0-34.2)	17.0 (16.5-17.5)	7.0 (6.6-7.4)	2.7 (2.4-3.0)	0.8 (0.6-1.0)	27.1 (27.0-27.2)
Some college or associate’s degree	43.1 (42.5-43.6)	44.7 (44.1-45.4)	59.6 (59.0-60.3)	18.0 (17.4-18.5)	11.1 (10.6-11.6)	2.1 (1.8-2.4)	30.9 (30.8-30.9)
Bachelor’s degree	42.0 (41.4-42.5)	7.5 (7.2-7.8)	17.4 (16.9-17.9)	31.4 (30.7-32.1)	25.8 (25.2-26.5)	1.8 (1.5-2.1)	20.4 (20.3-20.5)
Master’s degree or higher	10.3 (10.0-10.7)	1.5 (1.4-1.7)	2.8 (2.6-3.0)	34.5 (33.8-35.2)	22.8 (22.2-23.5)	2.4 (2.1-2.8)	8.2 (8.2-8.3)
Doctorate degree^c^	2.6 (2.4-2.8)	1.2 (1.1-1.3)	1.6 (1.4-1.7)	7.8 (7.4-8.2)	36.9 (36.1-37.6)	92.6 (92.0-93.2)	2.9 (2.9-3.0)
Income							
0-$40 000 or loss^d^	5.3 (5.1-5.6)	37.7 (37.7-38.3)	16.8 (16.3-17.3)	16.8 (16.3-17.4)	6.2 (5.8-6.6)	3.5 (3.1-3.9)	21.1 (21.0-21.2)
$40 001-$75 000	24.8 (24.2-25.3)	34.0 (34.0-34.6)	34.5 (33.9-35.2)	31.2 (30.5-31.8)	18.2 (17.6-18.8)	10.0 (9.3-10.6)	30.9 (30.8-31.0)
$75 001-$125 000	39.7 (39.2-40.3)	20.7 (20.7-21.2)	33.0 (32.4-33.6)	32.6 (31.9-33.3)	33.0 (32.3-33.7)	13.8 (13.1-14.5)	28.7 (28.7-28.8)
>$125 000	30.2 (29.6-30.7)	7.5 (7.5-7.8)	15.7 (15.2-16.2)	19.4 (18.8-19.9)	42.6 (41.9-43.4)	72.8 (71.8-73.7)	19.3 (19.2-19.4)
Residence							
Urban	74.2 (73.7-74.6)	77.6 (77.2-78.1)	74.1 (73.5-74.6)	81.1 (80.6-81.6)	76.9 (76.3-77.6)	83.4 (82.7-84.2)	76.6 (76.5-76.6)
Rural	25.8 (25.4-26.3)	22.4 (21.9-22.8)	25.9 (25.4-26.5)	18.9 (18.4-19.4)	23.1 (22.4-23.7)	16.6 (15.8-17.3)	23.4 (23.4-23.5)

^a^ Numbers in column headings are based on unweighted numbers and rounded following US Census rules. Data are from the Mortality Disparities in American Communities, limited to adults 26 years or older who were employed at time of the American Community Survey administration. The percentages are based on weighted data.

^b^ Includes American Indian or Alaska Native, Asian, Native Hawaiian or Other Pacific Islander, and more than 1 race.

^c^ Includes doctorate degree and postbaccalaureate professional degree beyond a BA.

^d^ Indicates negative or a loss in income dollars for the past 12 months.

**Table 2.** Age- and Sex-Standardized Rates and Adjusted Hazards Ratios of Alcohol-Related Deaths per 100 000 Person-Years by Occupational Group^a^

Occupational group^b^	Alcohol-related death rates per 100 000 person-years (95% CI)		*P* value^c^	AHR of alcohol-related death (95%CI)^d^
	Unadjusted	Age- and sex-standardized		
Registered nurses (n = 42 000)	9.0 (6.5-12.3)	16.8 (11.6-22.0)	.32	1.14 (0.83-1.56)
Health care support workers (n = 39 000)	11.9 (9.0-15.5)	19.5 (14.4-24.7)	.24	1.01 (0.77-1.33)
Health technicians (n = 32 500)	9.0 (6.2-12.6)	13.8 (9.1-13.8)	.10	0.75 (0.53-1.06)
Social and behavioral health workers (n = 27 000)	11.3 (7.8-15.8)	16.5 (10.9-22.1)	.36	1.17 (0.83-1.65)
Other diagnosing or treating clinicians (n = 22 500)	9.6 (6.0-14.5)	11.6 (6.8-16.4)	.02	1.13 (0.75-1.06)
Physicians (n = 13 000)	5.6 (2.4-11.0)	4.2 (1.3-7.1)	.0001	0.65 (0.32-1.34)
Non–health care workers, (n = 1 662 000)	18.6 (17.9-19.2)	18.1 (17.5-18.7)	NA	1.0

Abbreviations: AHR, adjusted hazard ratio; NA, not applicable.

^a^ Data from the Mortality Disparities in American Communities database and limited to adults 26 years or older who were employed at time of the American Community Survey.

^b^ Numbers in rows are based on unweighted numbers and rounded to 500s following Census rules. Non–health care workers represent the reference group.

^c^ Derived from Cox proportional hazards models controlling for age, sex, and an age × sex interaction term.

^d^ Models are mutually adjusted for occupational group, age, sex, race and ethnicity, marital status, educational level, residence (urban or rural), and income group.

## Discussion

Compared with non–health care workers, health care workers, especially physicians, had lower crude alcohol-related death rates. Following sociodemographic adjustment, however, significant group differences were no longer observed.

When viewed in relation to elevated drug overdose death rates for some health care worker groups,^2^ the alcohol-related mortality results suggest health care workers do not have a general underlying liability to substance-related deaths. Specific occupational factors, such as access to controlled medications, may pose drug overdose risks that do not extend to alcohol-related deaths. However, alcohol-related mortality represents an extreme end point and alcohol-related morbidity remains a common problem. Study limitations include termination of mortality data before the COVID-19 pandemic, misclassification of alcohol-related cause of death in death records,^1^ absence of key alcohol-related risk factors such as personal or family history of alcohol use, and an inability to measure occupational transitions during the 11-year follow-up.

The risks of alcohol-related deaths among health care workers did not exceed those of non–health care workers. However, this finding does not diminish the importance of improving management of alcohol-related problems among health care workers. Future research should compare problematic alcohol use among health care worker groups during the COVID-19 and post–COVID-19 periods.

## Notes

Supplement 1. **eTable 1.** *ICD-10-CM* Alcohol-Related Death Codes **eTable 2.** Health Care Worker Groups and Constituent Occupations (Occupation Codes)

Supplement 2.
Data Sharing Statement
