# Supplementary material for: Alcohol-Related Deaths of US Health Care Workers
Source: JAMA Netw Open. 2024 May 8;7(5):e2410248. doi: 10.1001/jamanetworkopen.2024.10248 (PMC11079688; doi:10.1001/jamanetworkopen.2024.10248)
Supplement: Supplement 1. — eTable 1. ICD-10-CM Alcohol-Related Death Codes eTable 2. Health Care Worker Groups and Constituent Occupations (Occupation Codes) [file jamanetwopen-e2410248-s001.pdf]

Supplementary Online Content

Olfson M, Cosgrove CM, Wall MM, Blanco C. Alcohol-related deaths of US health care workers. *JAMA Netw Open*. 2024;7(5):e2410248.
doi:10.1001/jamanetworkopen.2024.10248

eTable 1. *ICD-10-CM* Alcohol-Related Death Codes

eTable 2. Health Care Worker Groups and Constituent Occupations (Occupation Codes)

This supplementary material has been provided by the authors to give readers additional information about their work.

© 2024 Olfson M et al. *JAMA Network Open*.

eTable 1. *ICD-10-CM* Alcohol-Related Death Codes

**Category**	**ICD-10-CM Codes**
Alcohol overdose	R78.0, T51.0,[A] T51.9,[A] X45, X65, Y15
Alcohol mental disorders	F10.1-F10.9
Alcohol-associated liver diseases	K70.0, K70.1, K70.2, K70.3, K70.4, K70.9
Alcohol-induced causes	E24.4, G31.2, G62.1, G72.1, I42.6, K29.2, K85.2, K86.0

Codes from White AM, Castle JP, Powell PA, Koob GF. Alcohol-related deaths during the COVID-19 pandemic. *JAMA* 2022;327(17):1704-1706.

[A] These two T codes used for multiple cause of death only

© 2024 Olfson M et al. *JAMA Network Open*.

**eTable 2.** Health Care Worker Groups and Constituent Occupations (Occupation Codes)	
**Groups**	**Constituent Occupations**
Physicians	Physicians and Surgeons (3060)
Registered nurses	Registered Nurses (3130)
Other diagnosing or treating clinicians*	Chiropractors (3000), Dentists (3010), Dietitians and Nutritionists (3030), Optometrists (3040), Pharmacists (3050), Physician Assistants (3110), Podiatrists (3120), Audiologists (3140), Occupational Therapists (3150), Physical Therapists (3160), Radiation Therapists (3200), Recreational Therapists (3220), Respiratory Therapists (3220), Speech-Language Pathologists (3230), Other Therapists (3240), Other Diagnosing and Treating Practitioners (3260)
Health technicians	Clinical Laboratory Technologists and Technicians (3300), Dental Hygienists (3310), Diagnostic Related Technologists and Technicians (3320), Emergency Medical Technicians and Paramedics (3400), Health Diagnosing and Treating Practitioner Support Technicians (3410), Licensed Practical and Licensed Vocational Nurses (3500), Medical Records and Health Information Technicians (3510), Dispensing Opticians (3520), Miscellaneous Health Technologists and Technicians (3530), Other Healthcare Practitioners and Technical Occupations (3540)
Health care support workers	Nursing, Psychiatric, and Home Health Aides (3600), Occupational Therapist Assistants and Aides (3610), Physical Therapist Assistants and Aides (3620), Massage Therapists (3630), Dental Assistants (3640), Medical Assistants and Other Healthcare Support Occupations (3650)
Social or behavioral health workers	Social Workers (2010), Psychologists (1820), Counselors (2000), Miscellaneous Community and Social Service Specialists (2020).
United States Bureau of Labor Statistics, 2000 Standard Occupational Classification (SOC) Users Guide. https://www.bls.gov/soc/2000/home.htm. *Excludes veterinarians.	

© 2024 Olfson M et al. *JAMA Network Open*.
