# Supplementary material for: Alcohol-Related Deaths of US Health Care Workers
Source: JAMA Netw Open. 2024 May 8;7(5):e2410248. doi: 10.1001/jamanetworkopen.2024.10248 (PMC11079688; doi:10.1001/jamanetworkopen.2024.10248)
Supplement: Supplement 2. — Data Sharing Statement [file jamanetwopen-e2410248-s002.pdf]

Data Sharing Statement

Olfson. Alcohol-Related Deaths of US Health Care Workers. *JAMA Netw Open*. Published May 08, 2024. doi:10.1001/jamanetworkopen.2024.10248

Data
Data available: Yes
Data types: Participant data with identifiers, Data dictionary
How to access data: Instructions for accessing data are available at https://www.census.gov/topics/research/mdac.Data_Availability.html#list-tab-1414305400
When available: beginning date: 01-01-2018

Supporting Documents
Document types: None

Additional Information
Who can access the data: Researchers who receive approval from the MDAC Steering Committee
Types of analyses: Research
Mechanisms of data availability: After approval of a research proposal
Any additional restrictions: Only results approved by the Census Data Review Board will be released.
